# Association between Entamoeba histolytica infection and human leukocyte antigen HLA- DRB1

**DOI:** 10.1016/j.amsu.2018.10.019

**Published:** 2018-10-18

**Authors:** Israa Mohammad Abd AL-Khaliq, Batool Mutar Mahdi

**Affiliations:** Al-Kindy College of Medicine, University of Baghdad, Iraq

**Keywords:** *Entamoeba histolytica*, HLA, Amebiasis

## Abstract

**Background:**

*Entamoeba histolytica* is the parasitic amoeba which caused amebiasis in human and responsible of 100,000 deaths every year. There is currently no vaccine against this parasite. The innate and adaptive immunity are important in protection against infection.

**Aim of the study:**

To investigate the association between HLA-DRB1 and *Entamoeba histolytica* infection.

**Patients and methods:**

The study is a case-controlled consisted from thirty Iraqi Arab Muslims patients with *Entamoeba histolytica* infection. The patients were consulted medical city and AL-Karama hospital for the period between May 2016 till February 2017. The control groups were sex and age matched with patient study group, they were consisted of thirty Iraqi Arab Muslims healthy individuals. HLA-DRB1 was done by SSOP method.

**Results:**

A total of 30 patients with *Entamoeba histolytica* infection were participated in this study. Their ages were range from 21 to 55 years. Males were 83.3% and the rest were females. The other is 30 control group was sex and age matched with patient study group. There was an increased frequency of HLADRB1*03:0101 and *11:0101 in patients group compared to control group (P = 0.002, Odds ratio = 7.42, 95% CI:2.07 to 26.55) and (P = 0.01, Odd ratio = 4.29, 95% CI: 1.41 to 13.06) respectively.

**Conclusions:**

HLA-DRB1*03:0101 and HLA-DRB1*110,101 may have a role in susceptibility to amebiasis.

## Highlights

• *Entamoeba histolytica* is the parasitic amoeba which caused amebiasis in human and responsible of 100,000 deaths every year.
• To investigate the association between HLA-DRB1 and *Entamoeba histolytica* infection.
• The study is a cross -sectional consisted from thirty Iraqi Arab Muslims patients with *Entamoeba histolytica* infection. The patients were consulted medical city and AL-Karama hospital for the period between May 2016 till February 2017. HLA-DRB1 was done by ssop method.
• A total of 30 patients with *Entamoeba histolytica* infection were participated in this study. There was an increased frequency of HLADRB1*03:0101 and *11:0101 in patients group compared to control group.
• HLA-DRB1*03:0101 and HLA-DRB1*110,101 may have a role in susceptibility to amebiasis.

## 1 Introduction

Amebiasis is a disease of gastrointestinal tract caused by ingestion cyst of protozoan *Entamoeba histolytica*. It is more common in developing countries where there is a contamination of food and water with human feces [1]. Disease is caused by ingestion of cyst and after excystation, the tophozoites adhere to colonic epithelial cells by a galactose/*N* –acetylgalactosamine (GAL/GalNAc)-specific lectin and the first defense mechanism is secretory IgA that prevent this attachment [2]. The other mechanism involved in the pathogenesis is apoptosis by non-Fas and non–tumor necrosis factor (TNF)-α1 receptor pathway [3]. The trophozoites had the ability to invade and lyse the tissue by cysteine proteinases enzyme that mediate inflammation by amplification IL-1 and cleavage IgA, IgG, C3a and C5a [4]. The host also had a defense mechanism against this protozoal represented by cell mediated immunity which is important in limiting and preventing recurrence of the disease. The first line of defense mechanism is innate immunity which is glycosylated mucous (MUC2) that lines the epithelial cell of the intestine [5] and cellular barrier represented by neutrophils that recruited by microbiome-mediated recruitment via CXCR2 and macrophages [6]. Once this line failed so the second line of defence mechanism which is adaptive immunity will be stimulated ending in production of cytokines, T cells, eosinophils, IgE, TNF-alpha and IL-25 production against amebic colitis [7]. The clinical perspective to the pathophysiology to this protozoa is *E histolytica* induced mucin excocytosis which is mediated by VAMP8 which is important in mucosal innate host defence mechanism and the virulence factor cysteine proteinase 5 of *E histolytica* that dissolve mucosa barrier [8,9]. It also affects T-cell activity that depressed against the trophozoites [10]. The other immune defense mechanism is macrophages that kill *E histolytica* trophozoites by oxidative pathways, non oxidative pathways, and nitric oxide (NO). Macrophages processed the trophozoite antigens and presented to Tc cells by presentation the Ags on HLA-DR (class II) molecule to cytotoxic lymphocytes (CD8^+^) positive cells. The molecular genetics of HLA is highly polymorphic located on short arm of chromosome number 6 that consist of three classes (Class I, II and III) [11]. The cause of amebiasis was due to interaction between genetic and environmental factors. The most important genetic factor is HLA alleles that play an important role in infection by *Entamoeba histolytica* and *E. dispar* cyst passer [12]. Among HLA classes; class I and II genes, HLA-class II*DRB1 code for DR β chains has the highest degree of polymorphism, and play an important role in the immune responses to different individuals to different antigens. This genetic variability affects susceptibility to several diseases like *Entamoeba histolytica* infection by affecting the antibody responses and T cell CD4^+^ stimulation within acquired immunity [13]. Regarding effect of sex on *Entamoeba histolytica* infection, there is no significant difference between male and females as predisposing factor for infection [14]. Seropositivity to *E. histolytica* was associated with low socio-economic families, poor hygiene, poor education of the head of the family and low level of education [15].

The present study aimed to evaluate and encourage further understanding of the disease and initiates field study on HLA typing. It helps in identifying risk factors and predisposing HLA-alleles (DRB1) that involved in *Entamoeba histolytica* infection in a sample of patients of Arab descent in Iraq and in determining the possible outcome or prognosis of the disease based on underlying genetic predispositions.

### 1.1 Patients and methods

The study is a case-controlled planned by HLA Research Unit in Al-Kindy College of Medicine. The consent of medicinal morals board was obtained for participants in this study. The revision was accepted by the Al-Kindy College of Medicine and Al-Kindy Teaching Hospital and Medical city Hospital. The knowledgeable permission was obtained from patients. The Scientific and Ethical Committee of Al-kindy medical college and Medical City Hospital had approved and registered the study. Written informed consents were obtained from the patients and control normal blood donors.

The inclusion criteria were patients with bloody diarrhoea caused by *Entamoeba histolytica*. On the other hand the exclusion criteria were patients with diareahoea caused by bacteria like *Salmonella*, *Shigella*, *Cholera*, viral, food poisoning, allergic diarrhoea and other parasitic infestation.

Sample size was 30 that calculated with confidence level 95%, confidence interval 5% and population proportion 50%.

**Fig. 1:**
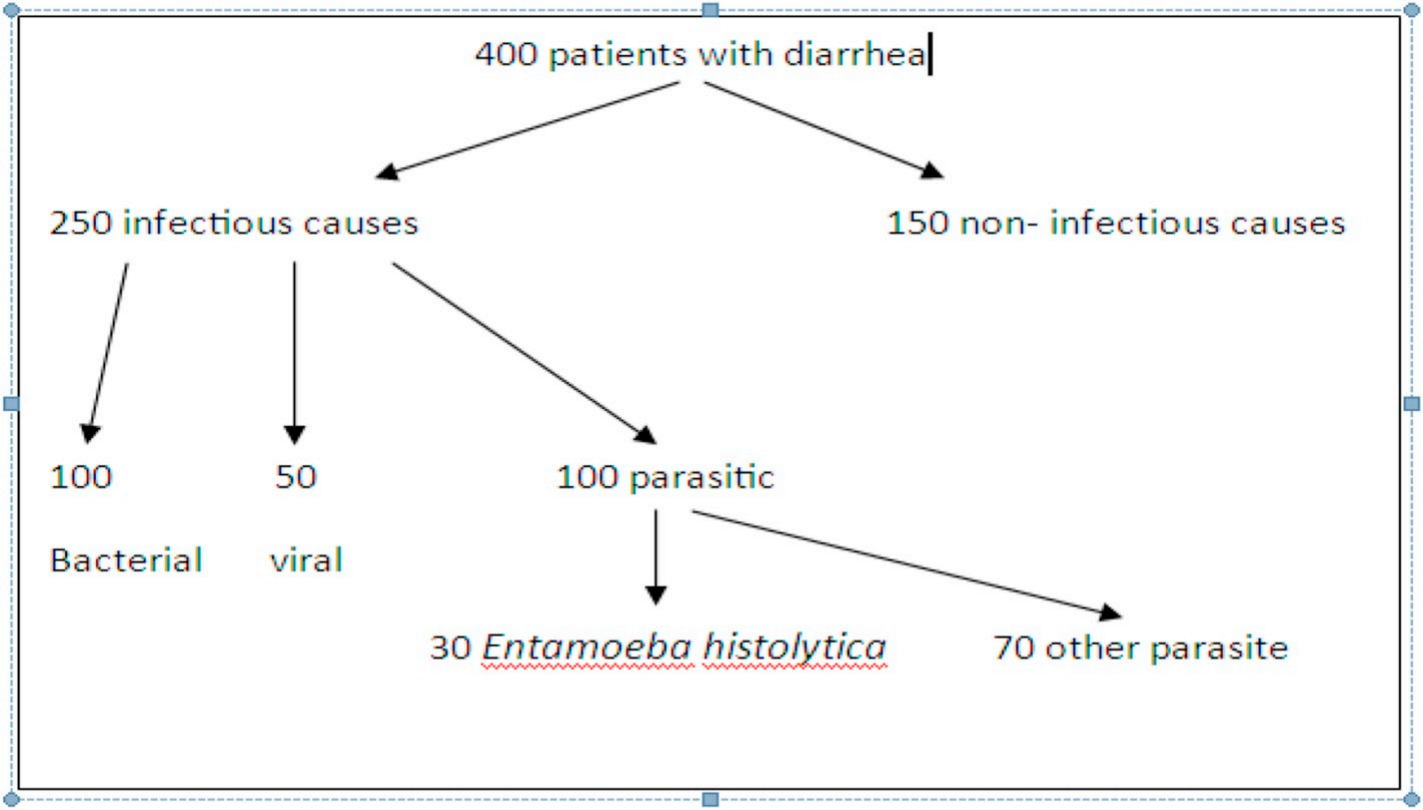
Flow diagram.

The flow diagram of the recruited participants was as follows (Figure-1-):

Data was collected from thirty patients of Arab descent in Iraq including demographic information age, sex, marital status, occupation, residential status. They were infected with *Entamoeba histolytica* as confirmed by stool examination and culture done and confirmed by Central Health Labrotary. They were diagnosed by confirming the presence of *Entamoeba histolytica* trophozoites or cyst in the stool and confirmed by culture. The patients were consulted medical city and AL-Karama hospital for the period May 2016 till February 2017. The questionnaire was obtained (name, age, address, and social history). The control groups were sex and age matched with patient study group; they were consisted of thirty Arab descents in Iraq healthy individuals.

Blood were obtained from the two groups and DNA was separated using DNA extraction by blood kit (QiAmp. DNA blood Mini Kit, Qiagen INC— Germany). DNA concentration and purity were determined using Nanodrop. Locus- and allele-specific amplification of genomic DNA was performed for DRB1 using sequence specific oligonucleotide probes (SSOP) using HLA-DRB1amplification and hybridization kits (SSO HLA type DRB1 plus and master mix for HLA type DRB1Amp. Plus kits -Innogenetics-Belgium). The procedure was done by automated method by AutoLipa 48 Innogenetics-Belgum. The results were interpreted using LiRas version-5.0 software- Innogenetics Belgium.

The primary outcome is diagnosis of patients with *Entamoeba histolytica* while the secondary outcome determination of HLA *DRB1 alleles that either predisposed or protective against infection with this parasite.

**Statistical analysis** was done using MiniTab version 3.0 software. The distribution of HLA alleles in patients and control groups were compared. In each comparison, the Odds ratio (OR) along with the 95% confidence interval (95% CI) was used. P-value less than 0.05 was considered statistically significant.

The work has been reported in line with the STROCSS criteria [16].

## 2 Results

A total of 30 patients with *Entamoeba histolytica* infection were participated in this study. Their ages were range from 21 to 55 years. Males were 83.3% and the rest were females. They were consisted of thirty Arab descents in Iraq healthy individuals. Their ages were range from 21 to 55 years. Males were 83.3% and the rest were females. The base lines characters' of the patients were shown in table −1-.

**Table 1:** Base line characters' of the patients.

No. = 30	Characteristic
31 ± 1.21	Age (X±SEM)
25 (83.3%)	Males
5(16.6%)	Females
27(90%)	Married
3(10%)	Single
30(100%)	Iraqi Nationality
8(26.6%)	Governmental Workers
22(73.3%)	Non Governmental Workers
2(6.6%)	Rural residency
28(93.3%)	Non Rural residency

HLA-DRB1* alleles using DNA based methodology (PCR-SSOP) were done for both of them. There was an increased frequency of HLA-DRB1*03:0101 and HLA-DRB1*11:0101 in patients group compared to control group (P = 0.002, Odds ratio = 7.42, 95% CI:2.07 to 26.55) and (P = 0.01, Odd ratio = 4.29, 95% CI: 1.41 to 13.06) respectively. Other alleles were detected either in patients group only or in control group only as shown in table −2-.

**Table-2:** Human leukocytes antigens (HLA-DRB1) alleles frequencies in patients with *Entamoeba histolytica* infection and healthy control group.

P- value	Odd ratio, (95% confidence interval)	control group No. = 30 No. %	patients group No. = 30 No. %	HLA-DRB1* alleles
na	na	2 (6.66)	0 (0)	02:0301
0.002	7.42 2.07 to 26.55	4 (13.33)	16 (53.33)	03:0101
na	na	2 (6.66)	0 (0)	03:0102
na	na	4 (13.33)	0 (0)	03:1701
na	na	1 (3.33)	0 (0)	03:1101
na	na	0 (0)	7 (23.33)	04:02:01
na	na	0 (0)	8 (26.66)	04:03:01
na	na	7 (23.33)	0 (0)	07:0101
na	na	2 (6.66)	0 (0)	08:0101
na	na	2 (6.66)	0 (0)	08:0201
0.010	4.29 1.41 to 13.06	7 (23.33)	17 (56.66)	11:01:01
na	na	0 (0)	1 (3.33)	11:01:03
na	na	0 (0)	1 (3.33)	11:33:01
na	na	4 (13.33)	0 (0)	11:03:01
na	na	4 (13.33)	0 (0)	11:67:01
na	na	2 (6.66)	0 (0)	12:09:01
na	na	2 (6.66)	0 (0)	13:05:01
na	na	7 (23.33)	0 (0)	13:18:01
na	na	2 (6.66|)	0 (0)	14:0101
na	na	8 (26.66)	0 (0)	14:02:01
na	na	0 (0)	8 (26.66)	15:01:01

na = not applicable.

Significant (03:01:01, 11:01:01).

## 3 Discussion

Amebiasis is the disease of the developing countries that cause dysentery, colitis and liver abscess. During infection with amebiasis, the host develops an immune response that is incapable of eliminating tissue resident parasites, while the parasite actively immunosuppresses the host immune response [17]. The existence of specific anti-Ia during *E. histolytica* infection induce immune disregulation [18]. Most individuals with symptomatic infections lead to dysentery while a minority progresses to chronic disease and liver amebiasis. HLA plays an important role in parasitic and protozoal infection because macrophage that phagocytosed the protozoal Ag will process it and present antigenic peptide to T cell that enhance the activation of immune response thus HLA plays an important role in control resistance or susceptibility to a disease development [19]. In this study, there was a significant increased frequency of HLA-DRB1*03:0101 and HLA-DRB1*11:0101 in patients group compared to control. Other study in Bangladeshi children demonstrated a potential protective association with the HLA class II allele DQB1*0601 and the heterozygous haplotype DQB1*0601/DRB1*1501. This relationship may explain the occurrence of amebiasis in some children who are exposed to the parasite and role of HLA class II immune responses in protection against *E. histolytica* infection while other alleles: DQB1*0202, DQB1*0301, and DRB1*0701 were not associated with amebiasis. Thus this studies showed the influence of human leukocyte antigen class II alleles on susceptibility to *Entamoeba histolytica* infection in Bangladeshi children [20]. Other study done in Mexico which is in agreement with our result, they found an association between HLA-DR3 and amebic liver abscess in Mexican mestizo adults and infants and no significant association with amebic rectocolitis was found. They also showed no specific HLA marker is associated with the asymptomatic cyst passers. So complication of amebiasis has also an association with HLA alleles as increased frequency of HLA-DR3 and complotype SCO1 in Mexican mestizo children with amoebic abscess of the liver [21]. Children with amoebic liver abscess revealed a significant increase in HLA-DR5, and the absence of HLA-DR6 when compared to adults with amoebic liver abscess, suggesting that in this ethnic group these class II HLA may contribute to pediatric amoebic liver abscess as contrasting to the adult type of this disease. HLA-DR3, HLA-DR5 and HLA-DR6 have all been associated with certain forms of immune-dysfunction, and may thus contribute to some of the clinical and immunological features of this parasitic disease. This discrepancy with our results may be due to criteria of the patients' selection which is in our study were complaining from bloody diarrhea of acute onset while in other studies either chronic condition or with liver abscess. Other important thing is ethnicity of our patients which they were Arab Muslims while in other studies their samples from different nations. Sample size is also an important factor in the study when sample size increased, it leads to increase the precision of our estimate but time and cost must be taken in consideration. Additionally, method used in detection HLA alleles and in our study highly expensive and costy molecular method used. Other factor is age of the patients which affect the immune response of the patients when increased leads to involution of the thymus and decrease immunity against infection. Lastly the specious of the *Entamoeba* whether *histolytica* or *E. dispar* is also affecting the relation with HLA alleles [22]. Thus, the role of HLA-DRB1 alleles is considered as a genetic predisposing factor to infection with *Entamoeba histolytica* like in this study, complications of this disease similar to liver abscess and potential predictor response to treatment as pharmacogenetic marker. Meanwhile, HLA-DRB1 alleles that identified in control group and not case patients were also important as a predictive genetic marker against infection with this parasite.

## 4 Conclusions

HLA may have a role in susceptibility to amebiasis, resistance to metronidazole treatment, chronicity of the disease and progression to cyst passer.

## Recommendations

Future researches need to be applied on large sample size and include other HLA class I and class II alleles.

## Ethical approval

Yes, Al-kindy college of medicine, University of Baghdad, 2017.

## Sources of funding

No.

## Author contribution

Israa did the test.

Batool wrote the paper.

## Conflicts of interest

No.

## Research registration number

3803.

## Guarantor

Consultant Dr Ali Ghalib.

## Funding

None.

## Provenance and peer review

Not commissioned, externally peer reviewed.
